# The *Cordyceps militaris*-Derived Polysaccharide CM1 Alleviates Atherosclerosis in LDLR^(-/-)^ Mice by Improving Hyperlipidemia

**DOI:** 10.3389/fmolb.2021.783807

**Published:** 2021-12-13

**Authors:** Fan Yin, Ping Lin, Wen-Qian Yu, Nuo Shen, Yuan Li, Shou-Dong Guo

**Affiliations:** Institute of Lipid Metabolism and Atherosclerosis, Innovative Drug Research Centre, School of Pharmacy, Weifang Medical University, Weifang, China

**Keywords:** bioactive polysaccharide, lipid homeostasis, hyperlipidemia, atherosclerosis therapy, pcsk9

## Abstract

Atherosclerotic cardiovascular disease has a high mortality worldwide. Our lab previously purified a polysaccharide designated as CM1 with (1→4)-β-D-Glc*p* and (1→2)-α-D-Man*p* glycosyls as the backbone. In this study, we investigated the anti-atherosclerosis effect of CM1 and the underlying mechanisms of action in a low-density lipoprotein receptor knockout (LDLR^(-/-)^ mouse model. It was found that CM1 significantly decreased the formation of atherosclerotic plaques. Mechanistically, CM1 enhanced plasma level of apolipoprotein A-I and decreased the plasma levels of triglyceride, apolipoprotein B, and total cholesterol. In the absence of LDLR, CM1 elevated the expression of very low-density lipoprotein receptor for liver uptake of plasma apolipoprotein B-containing particles and reduced hepatic triglyceride synthesis by inhibiting sterol regulatory element binding protein 1c. CM1 improved lipids excretion by increasing the liver X receptor α/ATP-binding cassette G5 pathway in small intestine. CM1 reduced lipogenesis and lipolysis by inhibiting peroxisome proliferator-activated receptor *γ* and adipose triglyceride lipase in epididymal fat. Furthermore, CM1 improved lipid profile in C57BL/6J mice. Collectively, CM1 can modulate lipid metabolism by multiple pathways, contributing to reduced plasma lipid level and formation of atherosclerotic plaques in LDLR^(−/−)^ mice. This molecule could be explored as a potential compound for prevention and treatment of hyperlipidemia and atherosclerosis.

## Introduction

Cardiovascular disease (CVD) is the leading cause of death in modern society, particularly in some developing countries (Torres et al., 2015; Zhao et al., 2019). Atherosclerotic CVD results in high mortality worldwide (Zhao et al., 2019). Hyperlipidemia is a lifestyle risk factor for atherosclerotic CVD and becomes a major cause of CVD in developing countries due to lack of preventive measures (Zhao et al., 2019). Cholesterol-lowering statins are the first-line drugs in prevention of CVD via inhibiting 3-hydroxy-3-methylglutaryl coenzyme A reductase (HMGCR). Although statins are typically well tolerated, accumulating evidence have demonstrated that they tend to induce muscle pain as well as other side effects (Schahbaz et al., 2018; Irvine, 2020). More importantly, CVD is not be completely eliminated by the current therapeutic strategies (Guo et al., 2020; Lin et al., 2021a). For this reason, researchers are prompted to search for new therapies for the prevention and treatment of CVD.

Accumulating evidence have demonstrated that natural compounds in foods of the daily human diet are important in preventing chronic non-communicable diseases, such as CVD (Ciecierska et al., 2019). Polysaccharide is one of these natural compounds, and its activity depends on the monosaccharide composition, molecular weight, glycosyl linkage pattern, degree of branching, and solubility. Two best-studied polysaccharides are *ß*-glucans and mannans. Recent studies indicate that *ß*-glucans, such as *ß*-1,3-, *ß*-1,4- and *ß*-1,6-linked glucans, have great potential in preventing CVD due to their hypocholesterolemic, immunomodulatory, anti-inflammatory, hypoglycemic, and anti-oxidative effects (Ciecierska et al., 2019; Hou et al., 2020; Korolenko et al., 2020). The hypolipidemic effect of *ß*-glucans are found to be related to its fermentation capability and viscosity (Slavin, 2005). Furthermore, entrapping the bile acid micelles can increase the elimination of cholesterol and fat (Lia et al., 1995). Mannans can act as immunomodulator as well as lipid-lowering agent. However, the mechanisms of lipid-lowering effect of the above polysaccharides are poorly studied (Korolenko et al., 2020). It was suggested that natural polysaccharides reduce cholesterol level via sterol regulatory element binding protein (SREBP)-2-HMGCR and bile acid synthesis pathways, and decrease triglyceride (TG) level by adipose triglyceride lipase (ATGL)-peroxisome proliferator-activated receptor (PPAR)α and SREBP-1c pathways (Wu et al., 2019; Yang et al., 2019; Yin et al., 2019; Yang et al., 2021). Recent studies also suggested that polysaccharides containing *ß*-glucans or mannan have a potential of ameliorating atherosclerosis (Dahech et al., 2013; Ciecierska et al., 2019; Korolenko et al., 2020).

*Cordyceps militaris* is an edible mushroom and is a traditional Chinese medicine, which has been used as tonics for centuries (Das et al., 2010; Panda and Swain, 2011; Dong et al., 2015). Natural *C. militaris* is very rare and expensive, artificially cultivated fungal fruity body of *C. militaris* is generally used in the modern society (Cui, 2015; Zhang et al., 2019b). Some medicinal and tonic products of *C. militaris* have been developed and commercialized around the world, especially in Asian countries (Panda and Swain, 2011; Cui, 2015; Dong et al., 2015; Zhang et al., 2019a). It is worth noting that the water extract of the fruity body of *C. militaris* has an anti-hyperlipidemic effect (Koh et al., 2003; Paterson, 2008; Kim et al., 2014; Tran et al., 2019). Polysaccharide is one of the major constituents in the water extract of *C. militaris* and has a variety of bioactive activities, such as anti-inflammation, anti-oxidation, anti-aging, and anti-tumor (Cui, 2015; Zhang et al., 2019b). However, most of the previous studies used crude extracts rather than purified polysaccharide of *C. militaris* in anti-hyperlipidemic research (Koh et al., 2003; Paterson, 2008; Kim et al., 2014; Tran et al., 2019). Presently, the lipid-lowering mechanisms of the polysaccharides from *C. militaris* are not clear. More importantly, little research has been carried out on mushroom-derived *ß*-glucans in their anti-atherosclerotic activity and mechanisms of action (Korolenko et al., 2020; Lin et al., 2021b).

Presently, apolipoprotein (apo) E-deficient (apoE^(-/-)^) and low-density lipoprotein receptor (LDLR)-deficient (LDLR^(-/-)^) mouse are two typical animal models for study of atherosclerosis. ApoE is a ligand of LDLR and LDLR-related protein (LRP) and serves for the clearance of chylomicrons and very low-density lipoprotein (VLDL) particles from circulation. Therefore, the mutation or absence of apoE can induce a complex lipoprotein metabolism and make it hard to explain the mechanisms of action of the tested compounds. Plasma apoB level is positively associated with CVD events (Khan et al., 2020). LDLR is a receptor for the uptake of circulating non-HDL particles via apoB100 (Veniant et al., 1998; Wouters et al., 2005). Upon LDLR deficient, VLDLR, SR-BI and LRP, can clear the apoB-containing lipoproteins via binding the truncated apoB48 and apoE (Wouters et al., 2005). Based on the previous studies, the first line drug simvastatin and fibrates could not reduce the high-fat diet-induced hyperlipidemia and the progress of atherosclerosis in apoE^(-/-)^ mice (Yin et al., 2019; Yang et al., 2021). Mechanistically, the clearance of plasma lipids by simvastatin need the presence of apoE (Wang et al., 2002), and fibrates seem to reduce the expression of hepatic scavenger receptor B type I (SR-BI), which is found to mediate the uptake of HDL as well as apoB-containing particles as a backup protein (Acton et al., 1996; Fu et al., 2003). Therefore, LDLR^(-/-)^ mouse is more suitable for evaluating the effect of lipid-lowering drugs than apoE^(-/-)^ mouse (Wouters et al., 2005).

In a previous study, we purified a polysaccharide CM1 from the fruity body of *C. militaris*. CM1 has a backbone of (1→4)-β-D-Glc*p* and (1→2)-α-D-Man*p* and a molecular weight of 700 kDa. CM1 possesses cholesterol efflux capacity *in vitro* (Hu et al., 2019). In this study, we investigate whether this polysaccharide CM1 can attenuate atherosclerosis *in vivo*. LDLR^(-/-)^ mice, whose lipid profiles is more comparable with human plasma (Wouters et al., 2005), was used to explore the effect of CM1 in attenuating atherosclerosis and its regulation on lipid metabolism-related genes and proteins.

## Materials and Methods

### Materials

The dried fruity bodies of *C. militaris* (L.) Link were cultured, identified and provided by professor Yanyou Su at Taishan Medical University. The fruity bodies of *C. militaris* were also deposited at School of Pharmacy of Weifang Medical University with an access number of 2013-03. Simvastatin and Oil Red O were the products of Sigma-Aldrich. Fenofibrate was purchased from Selleck (Shanghai, China). Rabbit polyclonal antibody against ATP-binding cassette (ABC) G5, mouse monoclonal antibodies against PPARα and γ, Niemann-Pick C1-like protein 1 (NPC1L1), ATGL, SREBP1c and 2, lipoprotein lipase (LPL), and VLDL receptor (VLDLR) were purchased from Santa Cruz Biotechnology (Santa Cruz, CA, United States). A rabbit monoclonal antibody against SR-BI, rabbit polyclonal antibodies against ABCG8 and liver X receptor *a* (LXRα) were from Abcam (Cambridge, MA, United States). Rabbit polyclonal antibodies against proprotein convertase subtilisin/kexin type 9 (PCSK9), albumin, and apoB were the products of Proteintech Group Inc. (CHI, United States). A mouse monoclonal antibody against glyceraldehyde-3-phosphate dehydrogenase (GAPDH) was purchased from Abbkine Inc. (Wuhan, China). A rabbit monoclonal antibody against hepatocyte nuclear factor 1α (HNF1α) was the product of Cell Signaling Technology Inc. (MA, United States). Secondary antibodies were bought from CWBIO (Beijing, China). Enhanced chemiluminescence (ECL) kits were bought from Thermo Scientific Pierce (Rockford, IL, United States). Assay kits for total cholesterol (TC) and triglyceride (TG) were purchased from Biosino Bio-technology and Science Incorporation (Beijing, China). Complete protease inhibitor cocktail tablets were bought from Roche (Schweiz, Germany). RIPA lysis buffer and bicinchoninic acid (BCA) protein quantitative kits were the products of Solarbio (Beijing, China). Double-deionized water was produced using a Milli-Q gradient system from Millipore (Bedford, MA). The remaining reagents used in this study were of the analytical grade.

### Preparation of the Polysaccharide CM1

**FIGURE 1:**
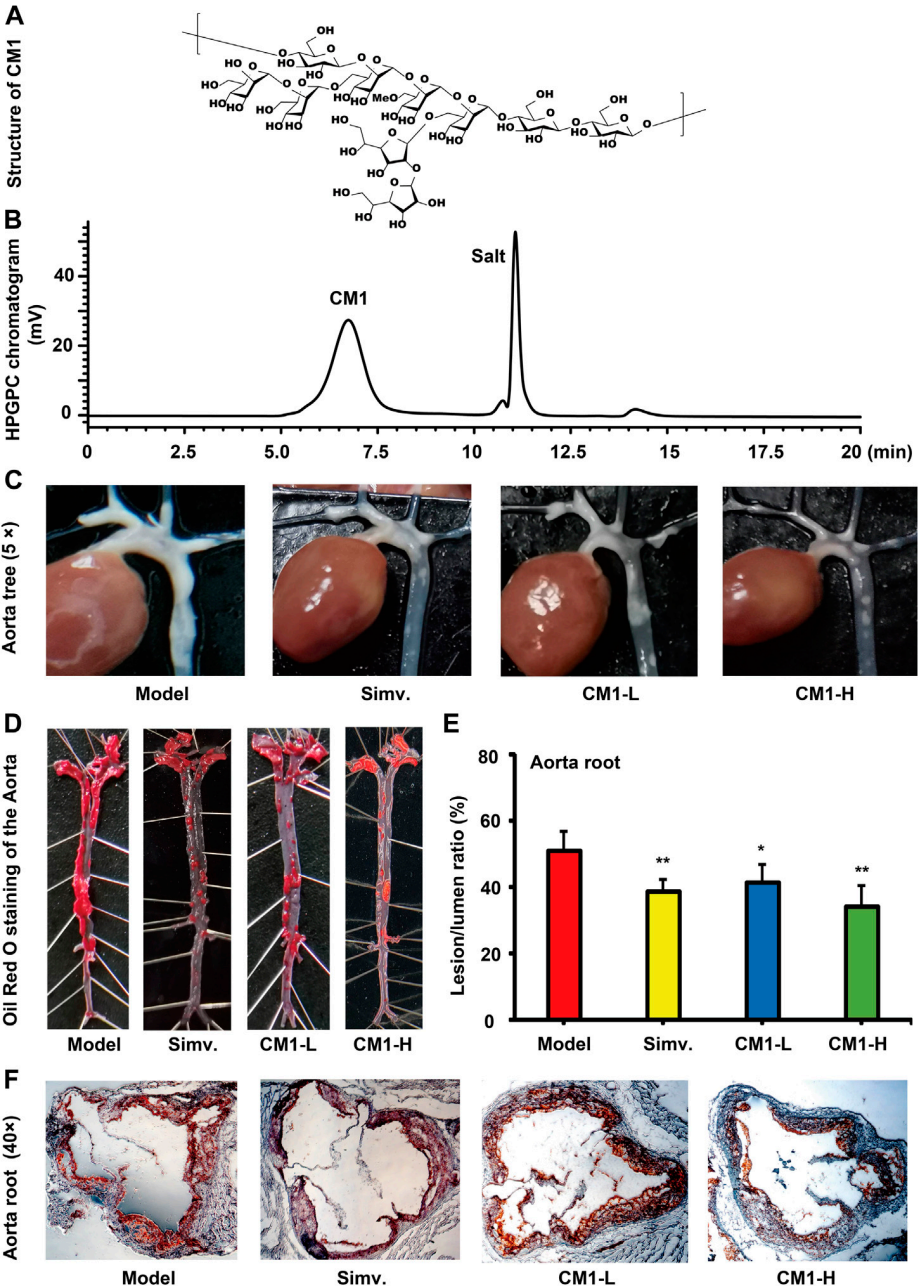
Structure and effect of polysaccharide CM1 on the formation of atherosclerotic plaques in the LDLR^(-/-)^ mice (*n* ≥ 6). **(A)**, structure of CM1; **(B)**, elution curve of CM1 on a TOSOH TSKgel G5000PWXL column (7.8 mm × 300 mm); **(C)**, representative images of the aortic tree; **(D)**, representative images of whole aorta after Oil Red O staining; **(E)**, statistical analysis of the lesion/lumen ratio in the aorta root; **(F)**, representative cross-sectional lesions of the aorta root. Data are presented as mean ± SD. Blank: chow diet group; Model: high-fat and–cholesterol diet group; Simv.: simvastatin; CM1-L: LDLR^(-/-)^ mice treated with CM1 at the dose of 25 mg/kg/d; CM1-H: LDLR^(-/-)^ mice treated with CM1 at the dose of 100 mg/kg/d. ^*^means *p* < 0.05 *vs*. Model, and ^**^means *p* < 0.01 *vs*. Model. All the abbreviations are applied for the rest of the figures.

In brief, the milled fruity bodies of *C. militaris* were defatted with 95% ethanol. The dried residues were extracted with eight volumes of distilled water at 95°C for 3 h. Crude polysaccharides were obtained via overnight ethanol precipitation at 4°C. Purified CM1 was obtained after anion exchange and exclusion chromatography as we previously reported (Hu et al., 2019). The same batch of CM1 with high purity was used in this study. The molecular weight of CM1 was evaluated as 700 kDa and its carbohydrate content was 99.3%. The presumed structure of CM1 was shown in Figure 1A. This polysaccharide was dissolved in 0.1 mol/L of Na_2_SO_4_ solution and its purity (Figure 1B) was determined by high performance gel permeation chromatography (HPGPC) with a RID-10A refractive index detector as previously described (Yang et al., 2021).

### Animal Grouping and Treatment

This study was approved by the laboratory animals’ ethical committee of Weifang Medical University and followed the NIH Guidelines for the Care and Use of Animals. LDLR^(-/-)^ mice (∼10 weeks old) with the gene background of C57BL/6J were purchased from GemPharmatech. (Jiangsu, China, license number: SCXK2018-0008). Several rounds of crossing were carried out to reproduce the LDLR^(-/-)^ mice. High-fat chow and regular chow were bought from Beijing HFK Bioscience Co., LTD (Beijing, China). After 1 week’s adaptive feeding, mice were randomly divided into four groups with eight mice in each group: the model group (Model, water by gavage), the simvastatin group (Simv., 50 mg/kg/d by gavage), the low-dose polysaccharide CM1 group (CM1-L, 25 mg/kg/d) and the high-dose polysaccharide CM1 group (CM1-H, 100 mg/kg/d). The dosage of the CM1 was determined according to our previous study (Guo et al., 2014). Mice were fed with a high-fat and -cholesterol chow (20% protein, 50% carbohydrate, 21% fat and 0.15% cholesterol). After 8 weeks of drug administration, the mice were weighed and sampled under anesthesia after overnight fasting. Before tissue sampling, the mice were perfused with 10 ml of PBS through left ventricle. The heart and aorta of mice in each group were removed and prepared for morphological staining or mechanistic studies. Epididymal fat was collected and the fat pad index ((fat weight × 100%)/body weight) was calculated according to our previous study (Yin et al., 2019).

Twenty-five C57BL/6J mice (∼11 weeks old) were provided by Beijing HFK bioscience Co., Ltd. (Beijing China). After 1 week’s adaptive feeding, the mice were randomly divided into five groups with five mice in each group: the blank control group, the model group, the fenofibrate group (Feno., 50 mg/kg/d by gavage), the low-dose polysaccharide CM1 group (CM1-L, 25 mg/kg/d) and the high-dose polysaccharide CM1 group (CM1-H, 100 mg/kg/d). Mice in the blank control group were fed with a regular chow, and the rest of mice were fed with the high-fat diet as described above. After 8 weeks intervention, the mice were sampled as describe above.

### Blood Analysis

Blood was collected from each mouse and centrifugated at 1,100 × *g* for 15 min at 4°C to obtain plasma. Plasma levels of TC and TG were evaluated using assay kits according to the manufacturers’ instructions. Furthermore, 150 μl of mixed plasma in each group was loaded onto a Superose™ 6 10/300 gel chromatography column and separated by ÄKTA fast protein liquid chromatography (FPLC) at 4°C. The column was eluted with normal saline at a flow rate of 0.3 ml/min 40 fractions were collected, and each fraction contained 0.5 ml eluate (Guo et al., 2016). 50 and 150 μl of the eluate in each fraction were assayed for TC and TG levels, respectively.

### Lipid Staining by Oil Red O

The whole aorta of the LDLR^(-/-)^ mouse was carefully isolated with the help of a stereomicroscope and photos of the aortic tree were captured with a camera. The whole aorta was then dissected and stained *en-face* with Oil Red O. In brief, the whole aorta was fixed with 4% paraformaldehyde for 2 h and immersed in 30% sucrose for another 2 h. Then, the intimal surface is exposed by a longitudinal cut through the inner curvature and down the anterior of the aorta (Daugherty et al., 2017). The aorta was washed with PBS and then kept in 60% isopropanol for 2 min. Staining was performed with the freshly prepared Oil Red O working solution consisting of 0.5% Oil Red O stocking solution (dissolved in isopropanol) and water in a ratio of 3:2 (v/v). Afterwards, the aorta was rinsed twice in 60% isopropanol to remove the non-specific binding Oil Red O. Images was captured by a camera.

Aorta root section (7 μm thickness) prepared by a cryostat (LEICA CM1850, Germany) with the presence of the aorta valve cups were collected and stained with oil red O. The sections were finally rinsed with distilled water, dried in the air, and mounted with glycerinum/PBS (9:1) (Guo et al., 2016; Yin et al., 2019). Lipid stained area was visualized using an Axio Vert. A1 microscope (Zeiss, Jena, Germany). Images were captured with an Axiocam 506 color camera (Zeiss) and quantified using Image-Pro Plus software (Version 6.0, Media Cybernetics, LP, United States).

### Protein Isolation, Electrophoresis, and Western Blotting

Total proteins from liver, whole aorta or small intestine were extracted and prepared for Western blotting according to our previous publications (Guo et al., 2016; Yin et al., 2019). In brief, the tissues (∼100 mg) were homogenized and lysed in RIPA lysis buffer with complete protease inhibitor for 30 min on ice. After centrifugation for 15 min at 20,000 × *g* at 4°C, supernatant was collected and assayed for protein concentration using the BCA method. For plasma, each 5 μl sample was mixed with 40 μl of normal saline and 15 μl 4 × loading buffer; the mixture was heated in a water bath at 80°C for 10 min. Equal amounts of protein (∼50 µg) or plasma medium (10 µL) were subjected to 10% or 4–20% gradient SDS-PAGE and transferred onto nitrocellulose membranes by electroblotting. Afterwards, the membranes were blocked in 5% nonfat dry milk for 2 h at room temperature, and then incubated with primary antibodies overnight at 4°C. After washing with PBS-T 3 times, the membranes were incubated with corresponding horseradish peroxidase-conjugated secondary antibodies for 2 h at room temperature. Immunoblots were revealed by enhanced chemiluminescence reaction and images were captured by Clinx ChemiScope 6000 pro (Shanghai, China), and densitometry analysis was conducted using Clinx Image Analysis Software (Shanghai, China). The expression of the proteins was normalized by housekeeping protein *ß*-actin (tissue part) or ponceau S staining (plasma part).

### Real-Time Quantitative PCR

For the isolation of total RNA, 50–100 mg of freezing sample was ground with liquid nitrogen in a pre-treated RNase free mortar. Total RNA was extracted from the ground sample with the classical Trizol method as we described in a previous article (Yang et al., 2019). The obtained total RNA was dissolved in 30–50 μl of RNase free water. The concentration and purity of the RNA were determined using a nanodrop UV spectrophotometer. In general, RNA was quantified for the following cDNA synthesis when the value of A260/A280 was above 1.90. cDNA was produced in an ABI Veriti 96 Well Thermal Cycler (Waltham, MA, United States) using FastQuant RT Kit from Tiangen Biotech Co., LTD. (Beijing, China). Real-time PCR was performed in an ABI QuantStudio3 PCR System (Waltham, MA, United States) using SYBR Green qPCR Master Mix and gene specific primers synthesized by Sangon Biotech (Shanghai, China). The program was set as following: initial denaturation at 95°C for 10 min followed by 40 cycles of 95°C for 15 s, 58°C for 30 s and 68°C for 60 s. The primers used in this study were listed in Table 1. The relative expression of target mRNA was calculated by normalizing target mRNA *C*_*t*_s to the housekeeping gene *GAPDH* (method of 2^*-DDCt*^) (Yang et al., 2019; Yin et al., 2019). Undetectable of the target mRNA was defined as the *C*_*t*_ value greater than 30 cycles.

**TABLE 1:** The primers used for the polymerase chain reaction (PCR) reaction.

Primer		Sequences (5–3′)
GAPDH	Forward	AGG​TCG​GTG​TGA​ACG​GAT​TTG
	Reverse	GGG​GTC​GTT​GAT​GGC​AAC​A
PPARγ	Forward	TCG​CTG​ATG​CAC​TGC​CTA​TG
	Reverse	GAG​AGG​TCC​ACA​GAG​CTG​ATT
PCSK9	Forward	GAG​ACC​CAG​AGG​CTA​CAG​ATT
	Reverse	AAT​GTA​CTC​CAC​ATG​GGG​CAA
SRB1	Forward	TGT​ACT​GCC​TAA​CAT​CTT​GGT​CC
	Reverse	ACT​GTG​CGG​TTC​ATA​AAA​GCA
PPARα	Forward	ATG​GTG​GAC​ACG​GAA​AGC​C
	Reverse	CGA​TGG​ATT​GCG​AAA​TCT​CTT​GG
LXRα	Forward	AGC​GTC​CAT​TCA​GAG​CAA​GT
	Reverse	CTC​GTG​GAC​ATC​CCA​GAT​CTC
SREBP-2	Forward	GGT​CAT​TCA​CCC​AGG​TCA​CA
	Reverse	TAC​CTG​GGA​GGA​TGT​CAC​CA
SREBP-1c	Forward	GAC​AGC​CCA​GTC​TTT​GAG​GA
	Reverse	GAG​AAG​CAC​CAA​GGA​GAC​GA
ACC-1	Forward	GAG​GTA​CCG​AAG​TGG​CAT​CC
	Reverse	GTG​ACC​TGA​GCG​TGG​GAG​AA
SCD-1	Forward	CAT​CAT​TCT​CAT​GGT​CCT​GCT
	Reverse	CCC​AGT​CGT​ACA​CGT​CAT​TTT
DGAT1	Forward	TGG​TGT​GTG​GTG​ATG​CTG​ATC
	Reverse	GCCAGGCGCTTCTCAA
DGAT2	Forward	GGC​TAC​GTT​GGC​TGG​TAA​CT
	Reverse	CAC​TCC​CAT​TCT​TGG​AGA​GC
FAS	Forward	CAT​CCA​CTC​AGG​TTC​AGG​TG
	Reverse	AGG​TAT​GCT​CGC​TTC​TCT​GC
ABCG5	Forward	ACT​GCT​TCT​CCT​ACG​TCC​TG
	Reverse	CTG​TAG​TTG​CCA​ATC​AGT​CGG

### Data Analysis

All the bioassay results were expressed as mean ± standard deviation (*SD*) for at least three independent experiments. Statistical analysis was performed by Student-T-Test. Differences were considered to be significant at a *p* < 0.05.

## Results

### CM1 Decreased Formation of Atherosclerotic Plaques in the LDLR^(-/-)^ Mice

Aortic tree is a common region of atherosclerotic lesions. There were clear atherosclerotic lesions in the model group. Oil Red O staining results confirmed the successful establishment of the atherosclerosis model in LDLR^(-/-)^ mice fed with the high-fat chow. Simvastatin and CM1 administration notably decreased the formation of atherosclerotic lesions in the aortic tree of the LDLR^(-/-)^ mice. The entire length of aorta from the heart to the iliac bifurcation was isolated and adventitial tissues were removed. Simvastatin and CM1 administration obviously decreased the formation of atherosclerotic lesions in the entire aorta (Figures 1C,D). Aortic root is the area with consistent presence of atherosclerosis in all pertinent models and the most common region for quantification of atherosclerosis (Daugherty et al., 2017). Oil Red O staining results revealed the percentage of atherosclerotic lesion in aortic root of the model, simvastatin, CM1-L and CM1-H administration group was 50.9, 38.6, 41.5, and 34.4%, respectively. Compared with the model group, simvastatin administration decreased the lesion formation by ∼24.2% (Figures 1E,F, *p* < 0.01), while CM1-L and CM1-H administration lowered the atherosclerotic lesions by ∼18.5% (*p* < 0.05) and 32.5% (*p* < 0.01), respectively. These results suggested that CM1 administration significantly reduced atherosclerotic plaque formation in a dose-dependent manner.

### CM1 Improved Plasma TG Profile of the LDLR^(-/-)^ Mice

**FIGURE 2:**
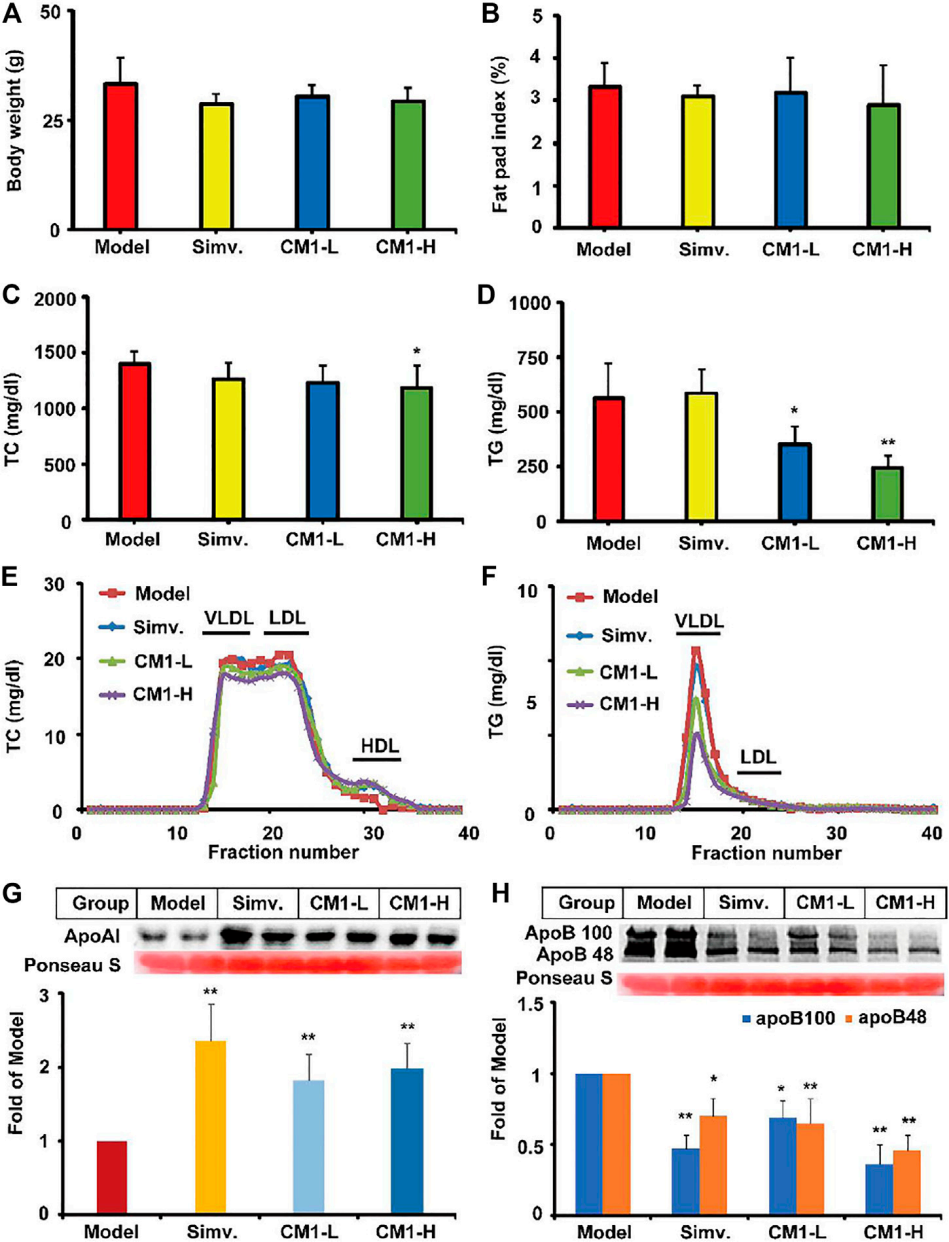
Effect of CM1 on the body weight, fat pad index, plasma lipid profiles and expression of apoAI and apoB (*n* ≥ 4). **(A)**, body weight of the mice in each group; **(B)**, fat pad index of the mice in each group. Fat pad index means the percentage of fat pad to body weight; **(C)**, plasma TC concentrations; **(D)**, plasma TG concentrations; **(E)**, plasma TC profiles of the lipoproteins after ÄKTA-FPLC separation; **(F)**, plasma TG profiles of the lipoproteins after ÄKTA-FPLC separation; **(G)**, plasma apoAI expression and densitometric quantification; **(H)**, plasma apoB expression and densitometric quantification. ^*^means *p* < 0.05 *vs*. Model, and ^**^means *p* < 0.01 *vs*. Model.

Although simvastatin and CM1 administration decreased the average body weight and fat pad index compared with the model group, there was no significant difference among groups (Figures 2A,B). As shown in Figures 2C,D, the plasma TC and TG levels in the model group were 1,403.1 ± 102.4 mg/dl and 562.3 ± 156.8 mg/dl, respectively. Simvastatin administration had no significant influence on the plasma TC and TG levels of the LDLR^(-/-)^ mice. It is worth noting that CM1 administration significantly decreased the plasma TC level at CM1-H group (Figure 2C, ∼19.5%, *p* < 0.05). Moreover, CM1 significantly decreased plasma TG level in a dose-dependent manner (Figure 2D, ∼37.4% reduction in CM1-H group, *p* < 0.01). Further ÄKTA FPLC analysis demonstrated that CM1 administration increased the high density lipoprotein cholesterol (HDL-C) level and mildly decreased the non-HDL-C levels (Figure 2E). CM1 administration notably decreased TG levels in the VLDL and LDL particle fractions in a dose-dependent manner (Figure 2F).

In this study, simvastatin administration significantly increased the protein level of apoAI (∼2.3-fold, *p* < 0.01) and decreased the protein level of apoB100 and apoB48 by ∼53% (*p* < 0.01) and ∼30% (*p* < 0.05), respectively, in the plasma of the LDLR^(-/-)^ mice (Figures 2G,H). CM1 increased the plasma level of apoAI (∼1.8-fold, *p* < 0.01, Figure 2G) and decreased the plasma level of apoB100 and apoB48 by ∼ 31 and 35% in CM1-L group, and ∼64 and 54% in CM1-H group (*p* < 0.01, Figure 2H), respectively. These results demonstrated that CM1 has comparable effect of simvastatin in improving apolipoprotein levels.

### CM1 Enhanced the Protein Expression of VLDLR and Decreased the Expression of SREBP-1c and ApoB Proteins in the Liver of LDLR^(-/-)^ Mice

**FIGURE 3:**
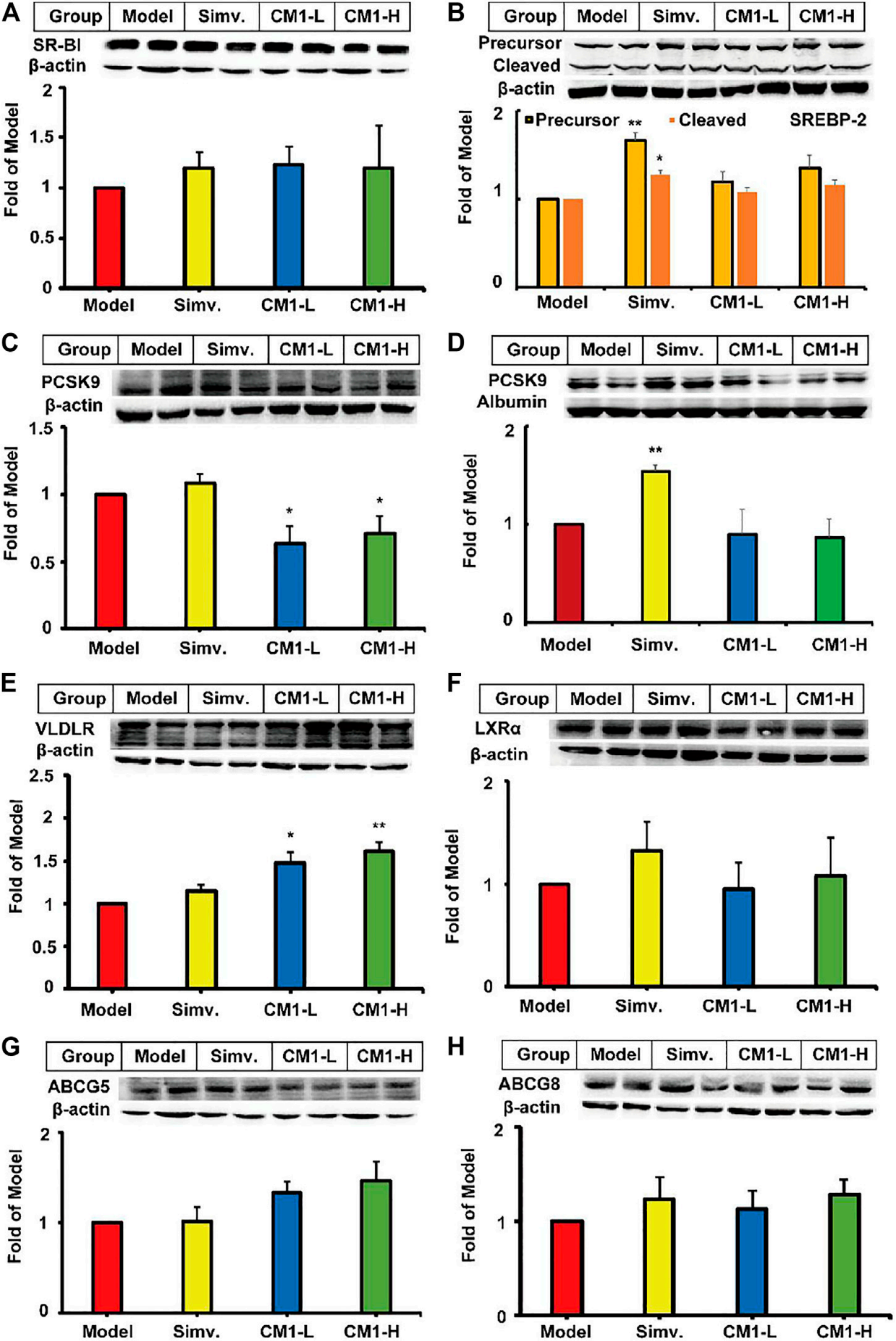
Effect of CM1 on TC metabolism related proteins in the liver and plasma of the LDLR^(-/-)^mice (*n* ≥ 4). Protein expression and densitometric quantification **(A)**, SR-B1 in the liver; **(B)**, SREBP-2 in the liver, precursor (∼125 kDa) and the cleaved mature form (∼68 kDa); **(C)**, PCSK9 in the liver; **(D)**, PCSK9 in the plasma; **(E)**, VLDLR in the liver; **(F)**, LXRα in the liver; **(G)**, ABCG5 in the liver; **(H)**, ABCG8 in the liver. ^*^means *p* < 0.05 *vs*. Model, and ^**^means *p* < 0.01 *vs*. Model.

SR-BI binds to HDL and promotes the reverse transport of excess cholesterol from peripheral tissues to the liver (Ma et al., 2020). In this study, simvastatin and CM1 administration had no effect on the protein expression of SR-BI (Figure 3A). SREBP-2 modulates the expression of genes, such as LDLR and PCSK9, involved in cholesterol metabolism at the transcriptional level (Li et al., 2020). Simvastatin administration had no significant effect on the expression of PCSK9, and VLDLR proteins (Figures 3C,E) in the liver. However, simvastatin increased the precursor SREBP-2 (∼125 kDa) and cleaved mature SREBP-2 (∼68 kDa) by approximately 66% (*p* < 0.01) and 28% (*p* < 0.05), respectively, compared to the model group (Figure 3B). Furthermore, simvastatin increased the plasma level of PCSK9 by approximately 54% compared to the model group (Figure 3D, *p* < 0.01). Interestingly, CM1 did not affect the expression of SREBP-2, but significantly decreased the expression of PCSK9 protein (*p* < 0.05) and increased the expression of VLDLR protein (∼47.0% in CM1-L group, and ∼61.0% in CM1-H group) in the liver of LDLR^(-/-)^ mice (Figures 3C,E). Furthermore, CM1 decreased the plasma level of PCSK9 by ∼13% compared to the model group at 100 mg/kg (Figure 3D). Both simvastatin and CM1 had no significant effects on the expression of LXRα, ABCG5 and ABCG8 proteins in the liver (Figures 3F–H).

**FIGURE 4:**
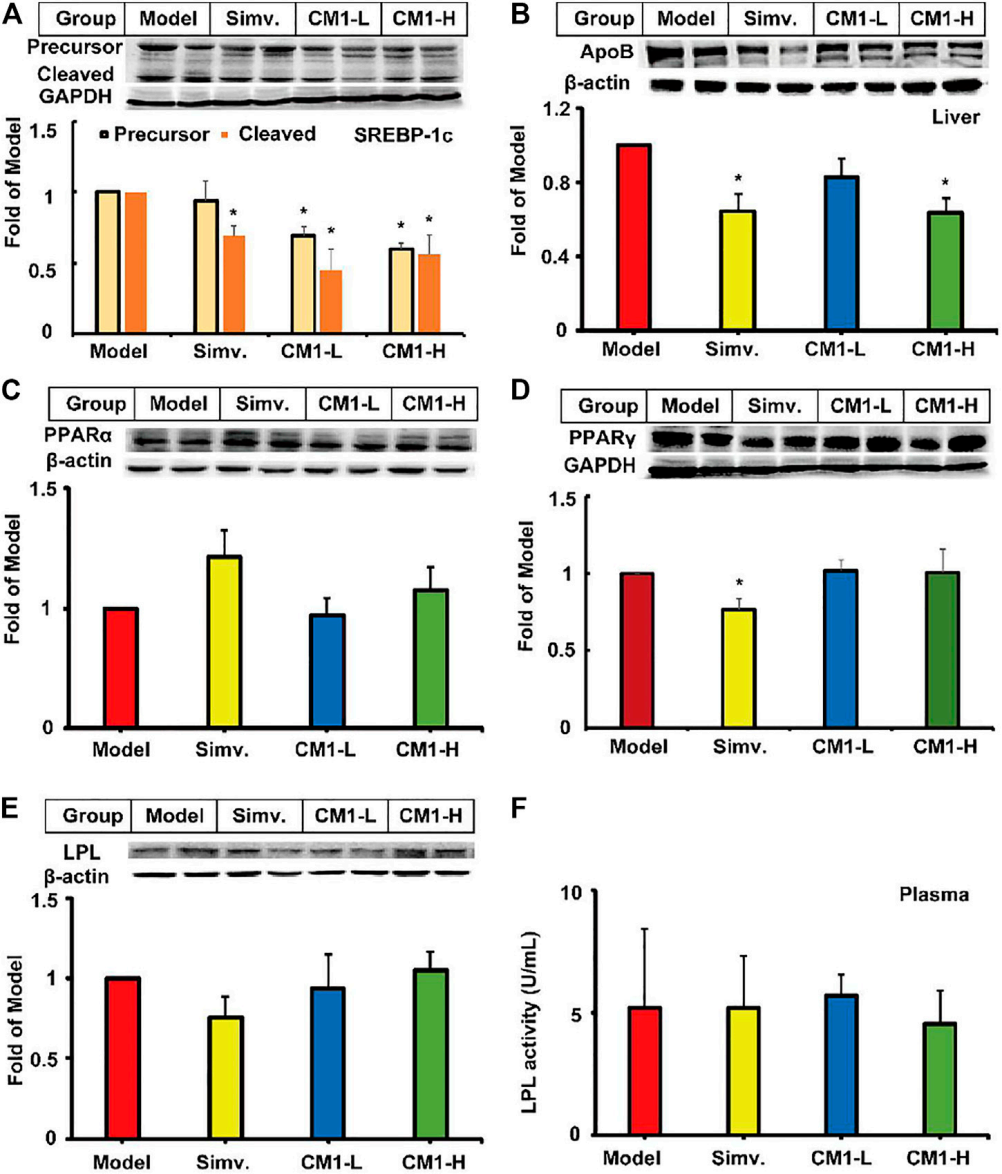
Effect of CM1 on TG metabolism related proteins in the liver and plasma of the LDLR^(-/-)^ mice (*n* ≥ 4). Protein expression and densitometric quantification. **(A)**, SREBP-1c, precursor (∼125 kDa) and cleaved mature form (∼68 kDa); **(B)**, apoB; **(C)**, PPARα; **(D)**, PPARγ; **(E)**, LPL; **(F)**, LPL activity in the plasma. ^*^means *p* < 0.05 *vs*. Model.

**FIGURE 5:**
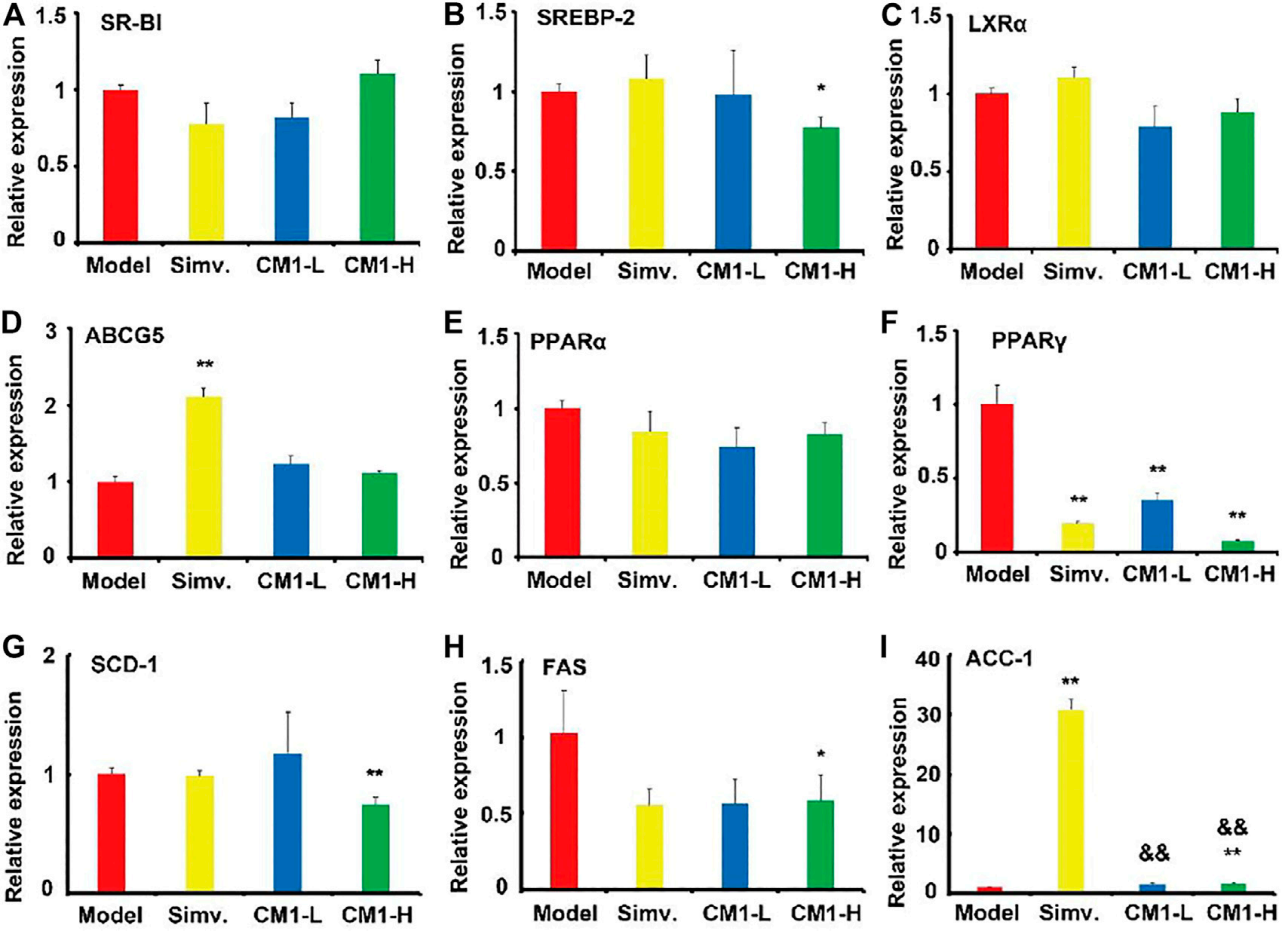
Effect of CM1 on the mRNA expression of lipid metabolism related genes in the liver of the LDLR^(-/-)^ mice (*n* = 3). **(A)**, SR-BI; **(B)**, SREBP-2; **(C)**, LXRα; **(D)**, ABCG5; **(E)**, PPARα; **(F)**, PPARγ; **(G)**, SCD-1; **(H)**, FAS; **(I)**, ACC-1. ^*^means *p* < 0.05 *vs*. Model, and ^**^means *p* < 0.01 *vs*. Model; ^&&^means *p* < 0.01 *vs*. Simvastatin.

Given the significant reduction of plasma TG levels in CM1 administration groups, we investigated the effect of CM1 on the expression of proteins related to TG metabolism. As shown in Figure 4, simvastatin had no significant effect on the expression of PPARα and LPL proteins in the liver. However, it reduced the expression of PPARγ by ∼24% compared to the model group (Figure 4D). CM1 administration decreased the expression of SREBP-1c and apoB proteins at 100 mg/kg and showed significant difference compared to the model group (Figures 4A,B, *p* < 0.05). As shown in Figure 4F, both simvastatin and CM1 administration had no effect on the plasma LPL activity in the LDLR^(-/-)^ mice. Additionally, simvastatin and CM1 administration had no effect on the mRNA expression of SR-BI, LXRα, and PPARα in the liver of the mice (Figure 5). However, simvastatin significantly increased the mRNA expression of ABCG5 (*p* < 0.01, Figure 5D) and decreased the mRNA expression of PPARγ (*p* < 0.01, Figure 5F). CM1 administration significantly decreased the mRNA expression of SREBP-2 at 100 mg/kg (Figure 5B). Furthermore, CM1 administration dramatically decreased the mRNA expression of PPARγ (Figure 5F, *p* < 0.01) as that of simvastatin. Of note, CM1 intervention (100 mg/kg), but not simvastatin, significantly reduced the mRNA levels of stearoyl coenzyme A desaturase-1 (SCD-1) and fatty acid synthase (FAS) by approximately 26 and 42%, respectively, compared to the model group (Figures 5G,H). Furthermore, simvastatin significantly increased the mRNA expression of acetyl-CoA carboxylase 1 (ACC-1) by ∼30-fold (Figure 5I, *p* < 0.01). Although the high dosage of CM1 also notably increased the mRNA level of ACC-1 (∼67% increase), the effect was significantly lower compared to that of simvastatin (Figure 5I, *p* < 0.01). These results suggested that CM1 may reduce TG synthesis in the liver of mice. In this study, the mRNA levels of VLDLR, diacylglycerol O-acyltransferase (DGAT)-1 and 2, SREBP-1c and PCSK9 were undetectable because their *C*_*t*_ numbers were greater than 30.

### CM1 Improved the Expression of LXRα/ABCG5 Pathway in the Small Intestine of the LDLR^(-/-)^ Mice

**FIGURE 6:**
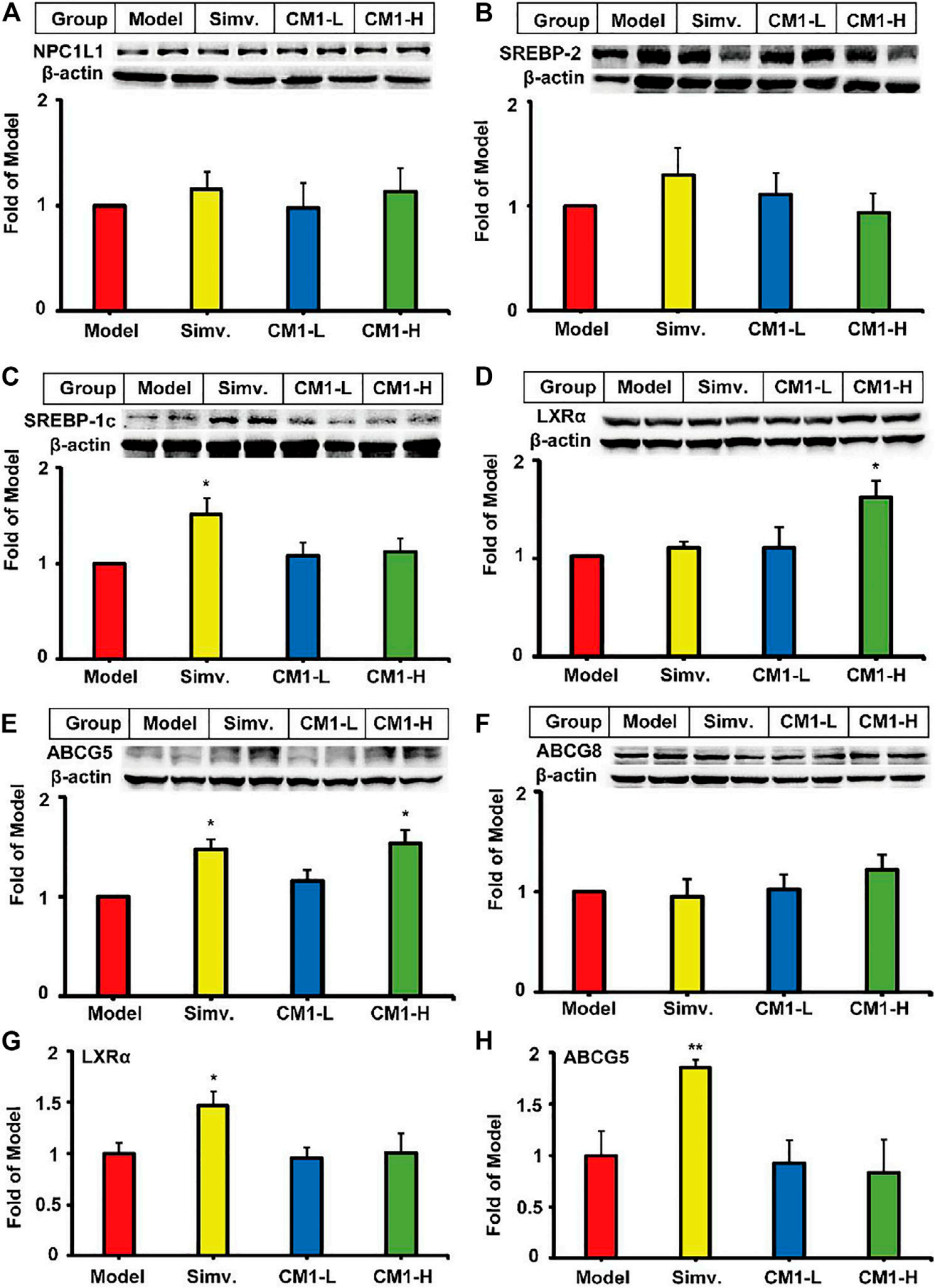
Effect of CM1 on the expression of lipid metabolism related genes and proteins in the small intestine of the LDLR^(-/-)^ mice (*n* ≥ 4). Protein expression and densitometric quantification. **(A)**, NPC1L1; **(B)**, SREBP-2; **(C)**, SREBP-1c; **(D)**, LXRα; **(E)**, ABCG5; **(F)**, ABCG8; **(G)**, mRNA expression of LXRα; **(H)**, mRNA expression of ABCG5. ^*^means *p* < 0.05 *vs*. Model, and ^**^means *p* < 0.01 *vs*. Model.

Small intestine plays a key role in lipid metabolism by modulating lipid absorption and excretion. Similar to simvastatin, CM1 administration had no effect on the expression of NPC1L1 and SREBP-2 proteins in the small intestine (Figures 6A,B). Simvastatin significantly increased the protein expression of SREBP-1c (Figure 6C, ∼50%, *p* < 0.05) and ABCG5 (Figure 6E, ∼47%, *p* < 0.05) compared to the model group. CM1 treatment significantly increased the protein expression of LXRα (Figure 6D, ∼61%, *p* < 0.05) and ABCG5 (Figure 6E, ∼53%, *p* < 0.05), but not ABCG8 (Figure 6F), at 100 mg/kg compared to the model group. Furthermore, simvastatin, but not CM1, significantly increased the mRNA expression of LXRα (∼1.5-fold, *p* < 0.05) and ABCG5 (∼1.8-fold, *p* < 0.01) in the small intestine of the LDLR^(-/-)^ mice (Figures 6G,H).

### CM1 Decreased the Expression of PPARγ and ATGL in the Epididymal Fat of the LDLR^(-/-)^ Mice

**FIGURE 7:**
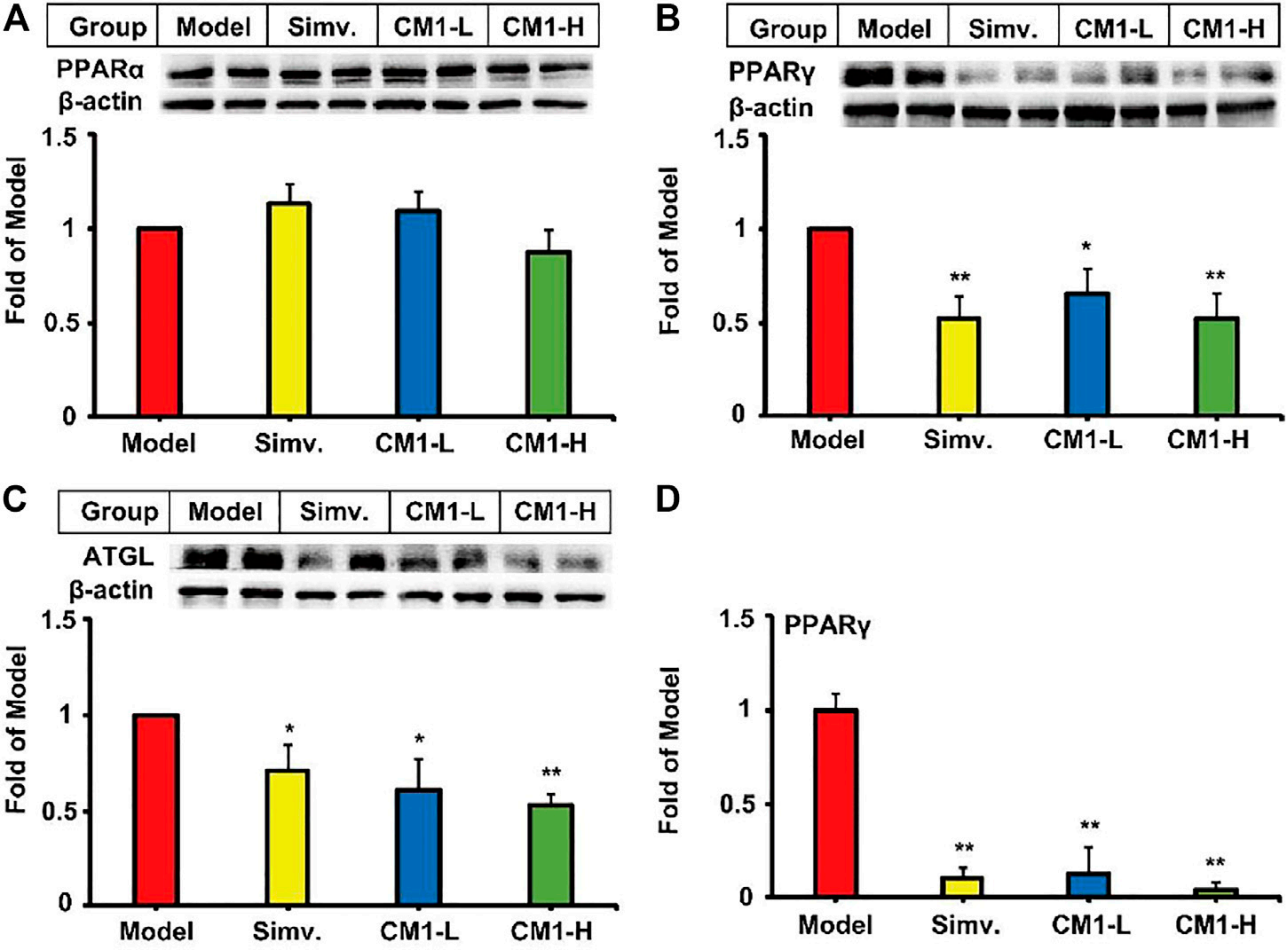
Effect of CM1 on the expression of lipid metabolism related genes and proteins in the epididymal fat of the LDLR^(-/-)^ mice (*n* ≥ 4). Protein expression and densitometric quantification. **(A)**, PPARα; **(B)**, PPARγ; **(C)**, ATGL; **(D)**, mRNA expression of PPARγ. ^*^means *p* < 0.05 *vs*. Model, and ^**^means *p* < 0.01 *vs*. Model.

Simvastatin or CM1 administration had no effect on the expression of PPARα (Figure 7A) in the epididymal fat. However, similar to simvastatin, CM1 intervention significantly down-regulated the expression of PPARγ (∼47% in CM1-H group, *p* < 0.01) and ATGL (∼45% in CM1-H group, *p* < 0.01) in a dose-dependent manner (Figures 7B,C). CM1 administration reduced the mRNA expression of PPARγ by ∼87% (*p* < 0.01) and ∼95% (*p* < 0.01) in the low dose and high dose group, respectively, as that of simvastatin (Figure 7D). The changes of the mRNA and protein of PPARγ were consistent in both simvastatin and CM1 intervention groups (Figures 7B,D). In this study, the mRNA levels of SREBP-1c and PPARα were undetectable because their *C*_*t*_ numbers were greater than 30.

### CM1 Improved the Lipid Profiles in C57BL/6J Mice

**FIGURE 8:**
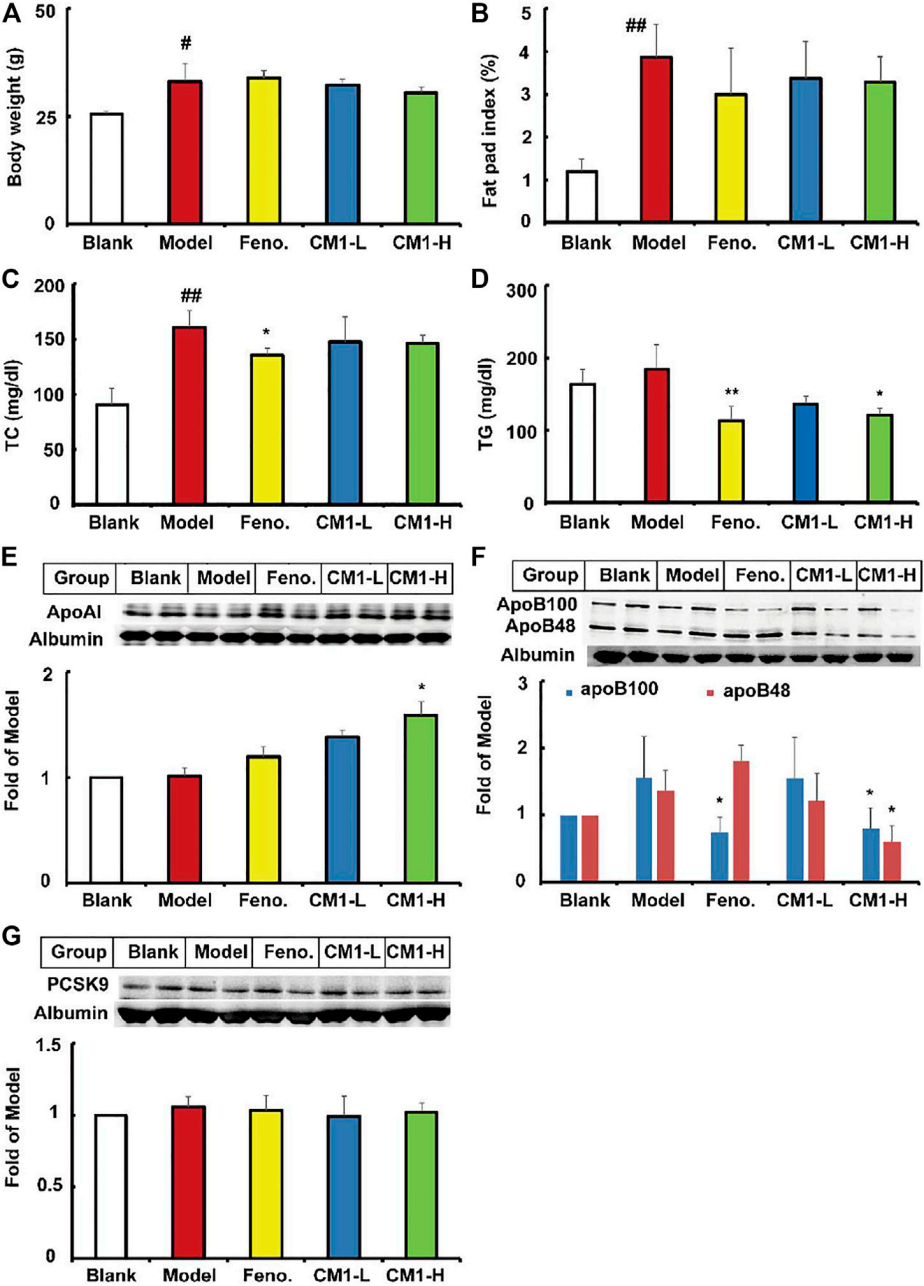
Effect of CM1 on the lipid profile and protein levels in the plasma of the C57BL/6J mice (*n* ≥ 4). **(A)**, body weight of the mice; **(B)**, fat pad index of the mice; **(C)**, plasma TC concentrations; **(D)**, plasma TG concentrations; **(E)**, plasma apoAI expression and densitometric quantification; **(F)**, plasma apoB expression and densitometric quantification; **(G)**, plasma PCSK9 expression and densitometric quantification. ^#^means *p* < 0.05 *vs*. Blank; ^##^means *p* < 0.01 *vs*. Blank; ^*^means *p* < 0.05 *vs*. Model, and ^**^means *p* < 0.01 *vs*. Model.

In this study, high-fat diet significantly increased the average body weight and the fat pat index by approximately 29.8% (*p* < 0.05) and 2.2-fold (*p* < 0.01), respectively, compared to the mice with regular chow diet (Figures 8A,B). Similar to the results in LDLR^(-/-)^ mice, CM1 or fenofibrate intervention had no effect on the average body weight or fat pad index compare to the model group. Furthermore, high-fat diet dramatically elevated the plasma TC level by 77.4% (*p* < 0.01), but not TG level, compared to the blank group (Figures 8C,D). In contrast to the model group, fenofibrate significantly reduced plasma TC level (∼16% reduction, *p* < 0.05) and TG level (∼38.8%), whereas CM1 significantly decreased plasma TG level (∼34% reduction, *p* < 0.01) but not TC level (Figures 8C,D). Of note, CM1 intervention (100 mg/kg) significantly increased the plasma level of apoAI by approximately 56% compared to the model group (Figure 8E, *p* < 0.05). In this study, fenofibrate significantly reduced the expression of apoB100 protein (∼52% reduction, *p* < 0.05), but not apoB48 protein, compared to the model group (Figure 8F). Furthermore, CM1 intervention (100 mg/kg) decreased the expression of apoB100 and apoB48 proteins by approximately 49 and 56%, respectively, compared to the model group (Figure 8F, *p* < 0.05). Both fenofibrate and CM1 intervention had no effect on the plasma level of PCSK9 (Figure 8G).

**FIGURE 9:**
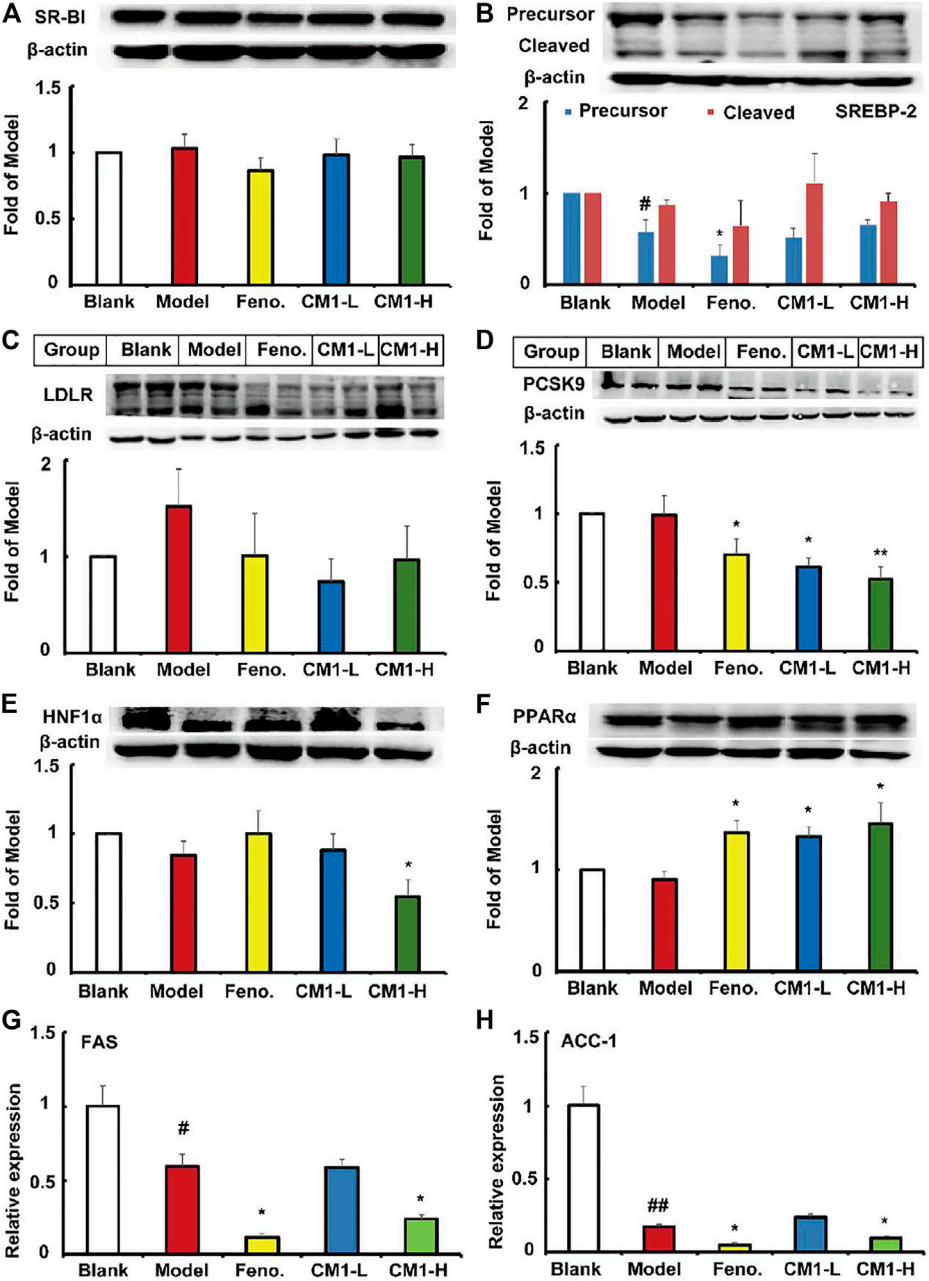
Effect of CM1 on the lipid metabolism related proteins in the liver of the C57BL/6J mice (*n* ≥ 3). Protein expression and densitometric quantification. **(A)**, SR-BI; **(B)**, SREBP-2; **(C)**, LDLR; **(D)**, PCSK9; **(E)**, HNF1α; **(F)**, PPARα; **(G)**, mRNA expression of FAS; **(H)**, mRNA expression of ACC-1. ^#^means *p* < 0.05 *vs*. Blank; ^##^means *p* < 0.01 *vs*. Blank; ^*^means *p* < 0.05 *vs*. Model, and ^**^means *p* < 0.01 *vs*. Model.

As shown in Figure 9A, high-fat diet and drug intervention had no effect on the expression of SR-BI protein in C57BL/6J mice. However, high-fat diet significantly decreased the expression of the precursor and mature SREBP-2 by approximately 43% (*p* < 0.05) and 24%, respectively, compared to the regular chow group (Figure 9B). Fenofibrate further decreased the expression of the precursor SREBP-2 by ∼45% (*p* < 0.05) compared to the model group. However, CM1 intervention had no effect on the expression of SREBP-2 (Figure 9B). Furthermore, high-fat diet and drug intervention had no effect on the expression of LDLR (Figure 9C). However, fenofibrate decreased the expression of PCSK9 protein by ∼28% (*p* < 0.05). CM1 intervention decreased the expression of PCSK9 protein by ∼38% (*p* < 0.05) and 47% (*p* < 0.01) in the low dose and high dose group, respectively, compared to the model group (Figure 9D). High-fat diet and fenofibrate intervention had no effect on the expression of HNF1α protein. Of note, the high dose CM1 (100 mg/kg) treatment reduced the expression of HNF1α protein by 45% compared to the model group (Figure 9E, *p* < 0.05). As a PPARα agonist, fenofibrate significantly increased the expression of PPARα protein by ∼51% compared to the model group (Figure 9F, *p* < 0.05). Similarly, CM1 intervention also significantly increased the expression of PPARα (*p* < 0.05). Finally, RT-qPCR experiments were performed to detect the expression of the genes related to TG synthesis in the liver of mice. As shown in Figures 9G,H, high-fat diet significantly reduced the mRNA expression of FAS and ACC-1 by ∼40% (*p* < 0.05) and 82% (*p* < 0.01), respectively, in the liver of C57BL/6J mice. Similar to the effects of fenofibrate, the high dosage CM1 intervention (100 mg/kg) dramatically decreased the gene expression of FAS and ACC-1 by approximately 60 and 43%, respectively, compared to the model group (Figures 9G,H, *p* < 0.05). In this study, the mRNA levels of SREBP-2, LDLR, PCSK9, and SCD-1 were undetectable because their *C*_*t*_ numbers were greater than 30.

## Discussion

LDLR^(-/-)^ mouse is one of the most extensively used models for the study of atherosclerosis (Getz and Reardon, 2016). Importantly, the lipid profiles of LDLR^(-/-)^ mouse is more comparable with human plasma and is more suitable for evaluating lipid-lowering drugs than apoE^(-/-)^ mouse model (Wouters et al., 2005). A high-cholesterol, high-fat diet can induce accumulation of LDL as well as VLDL in blood circulation. In LDLR^(-/*-*)^ mice, plasma cholesterol as well as TG is positively correlated with atherosclerotic lesion size, and HDL cholesterol is negatively correlated with the lesion size in the aortic root (Getz and Reardon, 2006; VanderLaan et al., 2009; Getz and Reardon, 2016). Simvastatin, a commonly used anti-hyperlipidemic drug in clinics, significantly decreased the formation of atherosclerotic plaques, but it had little effect on lipid profiles of the LDLR^(-/-)^ mouse. Our data were consistent with previous studies carried out by different groups (Chen et al., 2002; Golledge et al., 2010; Cheon et al., 2017). Furthermore, the effects of simvastatin on plasma apoAI and apoB were consistent with previous studies (Cheon et al., 2017; Song et al., 2011; Ma et al., 2015). Mechanistically, the anti-atherosclerotic effect of simvastatin can be mainly attributed to its anti-inflammatory and vascular repair functions in LDLR^(-/-)^ mice (Chen et al., 2002; Cheon et al., 2017).

The backbone of polysaccharide CM1 is consisted of (1→4)-β-D-Glc*p* and (1→2)-α-D-man*p*, branching at the *O*-6 positions of (1→2,6)-α-D-man*p* with (1→2) linked-β-D-gal*f*, (1→2) linked *a*-D-man*p* or methyl (Hu et al., 2019). In this study, we report the anti-atherosclerotic effect of CM1 in LDLR^(-/-)^ mouse. This effect of the water-soluble polysaccharide CM1 was consistent with previous studies, that demonstrated that the carbohydrates of *C. militaris* or *Cordyceps sinensis* (also belongs to the *Cordyceps* family) can reduce atherosclerotic lesions (Yamaguchi et al., 2000; Lin et al., 2021a). Another study also demonstrated that the alkaline-extracted *C. militaris* polysaccharide CM3II (∼19.1 kDa), which consisted of →4)-β-D-Man*p*(1→6)-α-D-Man*p*(1→6)-β-D-Man*p*(1→ glycosyls, can attenuate atherosclerosis in apoE^(-/-)^ mice (Yang et al., 2021). Furthermore, the polysaccharides from *Polygonatum sibiricum* showed hypolipidemic and anti-atherosclerotic effects in a rabbit model (Yang et al., 2015). Recent studies revealed that *P. sibiricum* polysaccharides are mainly composed of (1→4)-manno- and (1→4)-gluco-pyranosyl residues with potential of the *ß*-anomeric configuration (Yelithao et al., 2016; Wang et al., 2020b). These data suggest that polysaccharides containing *ß*-D-glycosyls and *a*-D-mannose may have an anti-atherosclerotic effect (Slavin, 2005; Ciecierska et al., 2019; Korolenko et al., 2020).

Accumulating evidence have demonstrated the positive correlation of plasma TC and the negative correlation of HDL-C with the incidence of atherosclerotic CVD (Guo et al., 2020; Lin et al., 2021b). Our data showed that CM1 administration reduced non-HDL cholesterol and increased plasma apoAI and HDL cholesterol levels. These results were in consistent with previous studies that water extracts of *Cordyceps* species have lipid-lowering and apoAI and HDL-C improving effects (Yamaguchi et al., 2000; Koh et al., 2003; Kim et al., 2014; Tran et al., 2019; Yang et al., 2021). A previous study also demonstrated that *C. militaris* polysaccharide RPS, which mainly composed of glucose (∼96%), has a hypolipidemic effect (Wang et al., 2015). The glucose of RPS may be in the *a*-D-configuration by comparison of its Fourier transform infrared spectrum with our recently published data of CM3I (Yang et al., 2021). Furthermore, the water extracted *C. militaris* polysaccharide CM1 showed a weaker TC-lowering effect in LDLR^(-/-)^ mice (∼19.5% reduction) compared to the alkaline-extracted *C. militaris* polysaccharide CM3II in apoE^(-/-)^ mice (∼42.7% reduction). This difference may be, at least in part, attributed to the distinct animal model. In this study, LDLR, a key receptor for clearance of non-HDL particles, is absent and there is no LDLR-mediated clearance of plasma cholesterol. Although the absence of LDLR ligand “apoE” reduces the binding of non-HDL particles with LDLR, LDLR still works for the clearance of apoB-containing particles in apoE^(-/-)^ mice (Murayama et al., 2000). ApoAI is the major apolipoprotein carried by HDL particles, and apoAI and HDL are two major acceptors of peripheral redundant cholesterol (Cucuianu et al., 2007). The elevated levels of apoAI and HDL-C may enhance the cholesterol accepting capacity of the plasma. Although CM1 had no effect on SR-BI, which is a key receptor for liver uptake of HDL particles (Ma et al., 2020), this molecule may increase SR-BI-mediated cholesterol clearance due to the increased apoAI and HDL-C levels in CM1 treatment group. Furthermore, CM1 administration significantly reduced the mRNA, but not protein, level of SREBP-2, which modulates cholesterol synthesis (Moslehi and Hamidi-Zad, 2018). This result was not consistent with the alkaline-extracted polysaccharide CM3II, which could significantly reduce the protein level of SREBP-2 in apoE^(-/-)^ mice (Yang et al., 2021). Additionally, the SREBP-2 results in the liver were consistent with the previous findings that simvastatin can up-regulate the expression of SREBP-2 in rats and C57BL/6J mice (Jia et al., 2014; Catry et al., 2015). In this study, simvastatin enhanced the levels of the precursor and mature SREBP-2, while a previous study demonstrated that statins mainly improve the level of the cleaved mature form rather than the precursor form of SPREBP-2 in HepG2 cells (Scharnagl et al., 2001). These inconsistent data may be induced by the distinct experimental model. Our data also demonstrated that CM1 showed no effect on NPC1L1-mediated cholesterol absorption and SREBPs-mediated lipid metabolism in the small intestine. Although CM1 have no effect on the excretion of cholesterol metabolites from liver, this molecule may increase lipid excretion from small intestine via activating LXRα/ABCG5 pathway through a post-translational regulation.

Recent studies have demonstrated that there is a positive correlation between plasma TG and CVD (Nordestgaard and Varbo, 2014; Tada et al., 2018; Vallejo-Vaz et al., 2020). The TG-lowering effect of CM1 was consistent with that of CM3II, which also contains *ß*-D-glycosyls and *a*-D-manno-pyranosyls (Yang et al., 2021). It was demonstrated that the *Trametes versicolor* polysaccharide, whose monosaccharide composition is similar to CM1, mainly composed of glucose, mannose and galactose, can also reduce TG level by ∼43.8% in hyperlipidemic mice (Huang et al., 2020). Furthermore, a previous study showed that *ß*-glucan is a key polysaccharide in the mushroom *Trametes versicolor* (Quayle et al., 2015). These results suggest that *ß*-D-glycosyls and *a*-D-manno-pyranosyls play key roles in TG and apoB metabolism. PCSK9, regulated by SREBP-2 at the transcriptional level, binds to LDLR family members (including VLDLR) and promotes their degradation in lysosome, contributing to the high non-HDL cholesterol and TG levels and the risk of CVD (Guo et al., 2020). In the absence of LDLR in LDLR^(-/-)^ mice, VLDLR may play an important role in the uptake of non-HDL particles, contributing to the clearance of cholesterol and TG in circulation. It is worth noting that CM1 administration significantly enhanced the expression of VLDLR, and this result was consistent with the decreased protein level of PCSK9. A previous study showed that *C. militaris* polysaccharide CM3-SII (∼25.2 kDa), whose backbone was composed of →4)-β-D-Man*p*(1→6)-β-D-Man*p*(1→6)-α-D-Man*p*(1→ glycosyls, can inhibit PCSK9 secretion *in vitro* (Wang et al., 2021). Therefore, the lipid-lowering effect of CM1 may be partially attributed to the elevated expression of VLDLR protein in the liver of the LDLR^(-/-)^ mice. Mechanistically, both SREBP-2 and HNF1α can modulate the expression of PCSK9. This study indicated for the first time that the polysaccharide CM1 from *C. militaris* may downregulate the expression of PCSK9 through HNF1α (Li et al., 2009).

Liver plays a key role in the assembly and secretion of apoB-containing VLDL particles, which carry the dominant TG in circulation. SREBP-1c regulates the expression of lipogenic genes at a transcriptional level, thereby modulating TG metabolism (Moslehi and Hamidi-Zad, 2018; Li et al., 2020). Our data suggested that CM1 reduced the level of genes related to TG synthesis, such as FAS and SCD-1. These results were consistent with the effects of the alkaline-extracted polysaccharide (mainly composed of glucose, galactose and mannose) from the edible mushroom *Amillariella mellea* (Yang et al., 2018). Several recent studies also demonstrated that heteropolysaccharides containing glucose, mannose and/or galactose can modulate the SREBP-1c pathway. For instance, the acidic heteropolysaccharide mainly composed of D-glucose, D-xylose, D-mannose, D-galacturonic acid and D-glucuronic acid from *Artemisia sphaerocephala* Krash seed can improve liver fatty acids via modulating hepatic SREBP-1c, SCD-1, ACC, and FAS expression (Zhang et al., 2019a). *Rosa roxburghii* Tratt fruit polysaccharide potentially composed of (1→5) linked-α-L-Ara*f*, (1→6) linked-α-D-Gal*p*, (1→3,4) linked-β-L-Fuc*p*, and (1→4) linked-β-D-Glc*p*, prevents hepatic steatosis via decreasing the expression of SREBP-1c, ACC-1 and FAS (Wang et al., 2018; Wang et al., 2020a).

PPARs are activated by a large variety of fatty acids and their derivatives. PPARα is a major inducer of fatty acid oxidation in liver, whereas overexpression of PPARγ in fat tissues is a major activator of adipocyte differentiation and energy storage in the form of TGs (Poulsen et al., 2012; Moslehi and Hamidi-Zad, 2018). In this study, CM1 administration had no effect on the expression of PPARα in the liver and epididymal fat. In contrast, CM1 significantly decreased the mRNA expression of PPARγ in the liver and epididymal fat and reduced the protein expression of PPARγ in the epididymal fat. PPARγ has been demonstrated to regulate the gene expression of VLDLR in adipocyte (Takazawa et al., 2009). However, the mRNA expression of PPARγ in the liver is very low as demonstrated previously (Poulsen et al., 2012) and the mRNA expression of VLDLR was undetectable in this study. Furthermore, multiple factors can influence the transcription and the post-transcription processes. Therefore, it is hard to explain the inconsistence between the reduced mRNA expression of PPARγ and the elevated expression of VLDLR protein in the liver of the LDLR^(-/-)^ mice. However, these data suggested that CM1 can inhibit TG storage and adipocyte differentiation. A previous study demonstrated that MDG-1, a *ß*-D-fructan polysaccharide, can inhibit PPARγ and activate PPARα in hyperlipidemic mice (Wang et al., 2017). The rice bran soluble polysaccharide mainly composed of *a*-1,6-glycosidic bonds can stimulate PPARα and inhibit PPARγ in mice that fed a high-fat diet (Nie et al., 2017; Chen et al., 2021). Furthermore, 1,6-α-glucans and 1,4-α-glucans also exhibited anti-atherosclerosis effects (Jin et al., 2014; Zhang et al., 2020). The okra polysaccharides, mainly rhamnogalacturonan, can improve metabolic disorders via down-regulating PPARγ (Fan et al., 2013; Liu et al., 2018). These data suggest that polysaccharides containing both *a*- and *ß*-glycosyls may have the capacity of inhibiting PPARγ. Furthermore, the modulation of PPARγ by the polysaccharide may be different in distinct animals. For example, the fucoidan from the brown seaweed *Ascophyllum nodosum* increased the protein expression of PPARγ in C57BL/6J mice and decreased its expression in apoE^(-/-)^ mice (Yin et al., 2019; Yang et al., 2019).

ATGL is highly expressed in white and brown adipose tissues and plays an important role in energy homeostasis (Schreiber et al., 2019). It initiates the hydrolysis of TGs to release fatty acids that are crucial energy substrates and precursors for the synthesis of membrane lipids (Ruiz-Leόn et al., 2019). Our data suggested that CM1 administration can decrease lipolysis by down-regulating ATGL expression. Therefore, CM1 treatment reduced the lipogenesis via down-regulating PPARγ and SREBP-1c and decreased lipolysis by inhibiting ATGL in epididymal fat. It was known that, PPARγ can regulate the expression of ATGL at the transcriptional level, and SREBP-1c transcription is decreased in ATGL deficient adipose tissue upon high-fat diet. These alterations may be explained as a metabolic compensatory mechanism (Schreiber et al., 2019). These complex mechanisms may have maintained the un-changed fat pad index in CM1 treatment group compared to the model group. The action of CM1 (∼700 kDa) on ATGL was different from the previous reported heteropolysaccharides from plants. For instance, polysaccharide (∼9.3 and 135 kDa) from *Cyclocarya paliurus* leaves, mainly composed of galactose, arabinose and rhamnose, can activate adipose ATGL in rats (Yang et al., 2016). The polysaccharide (∼8.5 kDa) from *Cichorium intybus* L. roots, mainly consisted of glucitol, fructose and glucose, improves ATGL expression in the liver of rats (Wu et al., 2018). Additionally, the alkaline-extracted polysaccharide (∼23.3 kDa, composed of 58.6% glucose, 19.8% galactose, 18.1% mannose, 3.3% glucuronic acid, and 1.5% fucose) from the edible mushroom *Amillariella mellea* can also activate ATGL in the adipose tissue in type 2 diabetic rats (Yang et al., 2018). These differences may be attributed to the different structural characteristics (such as molecular weight, glycosyl linkage pattern) of the polysaccharides and the distinct animal models.

LPL plays a central role in TG metabolism by hydrolysis of TGs in TG-rich lipoproteins (Benlian et al., 1996; Basu and Goldberg, 2020)). Our study showed that CM1 had no effect on the protein expression of LPL in the liver and the LPL activity in the plasma. The TG-lowering effect of CM1 was consistent with the downregulation of apoB in the plasma and liver. Mechanistically, the reduction of apoB may result from an enhanced intracellular degradation, a decreased synthesis and/or secretion of apoB, and a low rate of TG biosynthesis (Benoist and Grand-Perret, 1996). Our data suggested that CM1 may reduce hepatic TG synthesis by down-regulating SREBP-1c, FAS, SCD-1, PPARγ and ATGL, contributing to the reduced assembly and secretion of VLDL particles and the decreased level of apoB in the plasma. However, whether CM1 could directly reduce the production of apoB, VLDL and TG need to be clarified in future studies.

**FIGURE 10:**
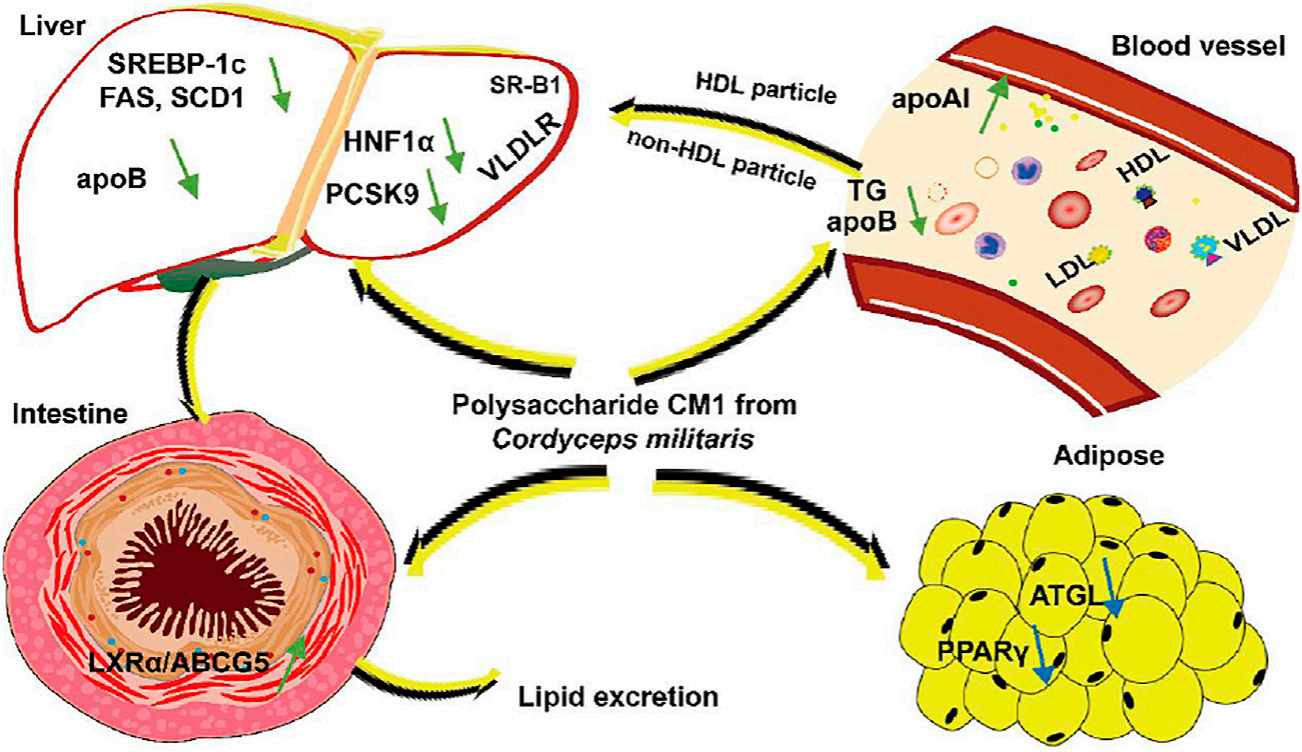
The proposed mechanisms of action of CM1 in mice.

Based on our current and previous data, the mechanisms of mushroom polysaccharides on modulating lipid metabolism are very complex. The structural characteristics, including molecular weight, monosaccharide composition, glycosyl linkage, configuration, and physiochemical properties, and even the animal models may have impact on their lipid-lowering activity. This study demonstrated for the first time that the polysaccharide CM1 from the fruiting body of *C. militaris* exhibited anti-atherosclerosis effect in LDLR^(-/-)^ mice via modulating multiple genes and proteins. The mechanisms of action were summarized in Figure 10. Firstly, CM1 may promote lipid profile via increasing the plasma apoAI level and decreasing apoB concentration. Secondly, it may decrease TG synthesis in the liver via down-regulating the expression of the related genes including FAS, ACC-1, and SCD-1. Thirdly, CM1 may reduce the expression of PCSK9 via decreasing HNF1α rather than SREBP-2. Finally, CM1 may improve lipid excretion from the small intestine via enhancing the LXRα/ABC transporter pathway. As the lipid profile of mouse is distinct from that of human, the effects of CM1 and the related mechanisms need to further investigated in other animal models such as hamster and rabbit (Gao et al., 2010). Furthermore, recent studies demonstrated that gut microbiota may also involve in lipid metabolism and atherosclerosis (Catry et al., 2015; Zhang et al., 2019a; Xu et al., 2020). Given the big molecular weight of CM1, this molecule is supposed to exert its function via modulating gut microbiota. Our next plan is to investigate whether *C. militaris* polysaccharides can regulate gut microbiota and the metabolites.

## Data Availability

The datasets presented in this study can be found in online repositories. The names of the repository/repositories and accession number(s) can be found in the article/Supplementary Material.

## Abbreviations

ABC, ATP-binding cassette; ACC, acetyl-CoA; apo, apolipoprotein; ATGL, adipose triglyceride lipase; CVD, Cardiovascular disease; DGAT, diacylglycerol O-acyltransferase; FAS, fatty acid synthase; FPLC, fast protein liquid chromatography; GAPDH, glyceraldehyde-3-phosphate dehydrogenase; HDL-C, high density lipoprotein cholesterol; HMGCR, 3-hydroxy-3-methylglutaryl coenzyme A reductase; HNF1α, hepatocyte nuclear factor 1α; LDL, low-density lipoprotein; LDLR, low-density lipoprotein receptor; LPL, lipoprotein lipase; LXRα, liver X receptor α; NPC1L1, Niemann-Pick C1-like protein 1; PCSK9, proprotein convertase subtilisin/kexin type 9; PPAR, peroxisome proliferator-activated receptor; SCD-1, stearoyl coenzyme A desaturase-1; SR-B1, scavenger receptor B type 1; SREBP, sterol regulatory element-binding protein; TC, total cholesterol; TG, triglyceride; VLDL, very low-density lipoprotein; VLDLR, very low-density lipoprotein receptor.
